# Supplementary material for: Antarctic glaciers export carbon-stabilised iron(II)-rich particles to the surface Southern Ocean
Source: Nat Commun. 2025 May 30;16:5015. doi: 10.1038/s41467-025-59981-y (PMC12125279; doi:10.1038/s41467-025-59981-y)
Supplement: Supplementary file 1 — Supplementary Information [file 41467_2025_59981_MOESM1_ESM.pdf]

Supplementary Information

Antarctic glaciers export carbon-stabilised iron(II)-rich particles to the surface Southern Ocean

Author list:

Rhiannon L. Jones[1,2*], Jon R. Hawkings[3,4], Michael P. Meredith[1], Maeve C. Lohan[2], Oliver W. Moore[5], Robert M. Sherrell[6], Jessica N. Fitzsimmons[6,7], Majid Kazemian[8], Tohru Araki[9], Burkhard Kaulich[8], Amber L. Annett[1]

*Corresponding author: rhines@bas.ac.uk

Affiliations:

1. British Antarctic Survey, Cambridge, UK
2. School of Ocean and Earth Science, University of Southampton, Southampton, UK
3. Department of Earth and Environmental Science, University of Pennsylvania, Philadelphia, PA, USA
4. iC3, Department of Geosciences, UiT, The Arctic University of Norway, Tromsø, Norway
5. Department of Environment and Geography, University of York, York, UK
6. Departments of Marine and Coastal Sciences and Earth and Planetary Sciences, Rutgers University, New Brunswick, New Jersey, USA
7. Department of Oceanography, Texas A&M University, College Station, Texas 77843, USA
8. Diamond Light Source Ltd., Harwell Science & Innovation Campus, Didcot OX11 0DE, UK
9. Institute for Molecular Science, Okazaki, Japan

Supplementary Figure S1: Scanning X-ray Microscopy (SXM) images and corresponding X-ray Absorption Near Edge Structure (XANES) and Near Edge X-ray Absorption Fine Structure (NEXAFS) spectra for Fe (green) and C (red). a) Image stack of material collected from King George Island at the Fe L-edge and C K-edge, with areas outlined in red that correspond to b) Fe L-edge XANES spectra. The relative abundance of Fe(II) and Fe(III) is quantified using the height of the two L-edge peaks, $L_{3\text{-}a}$ and $L_{3\text{-}b}$, as highlighted with the blue dotted lines. and c) C K-edge NEXAFS spectra. The three peaks annotated by the blue dotted line on this spectrum are at 285 eV (aromatic C), 288 eV (aliphatic/carboxylic), and 290.8 eV (inorganic carbonate). Panels d), e), f) show the equivalent maps and corresponding spectra for a set of particles collected and imaged, from Anvers Island. The three peaks annotated by the blue dotted lines in f) are at 285.2 eV (aromatic) and 288.2 eV (carboxylic) and 290.6 eV (inorganic carbonate). The y-axis is in arbitrary units.

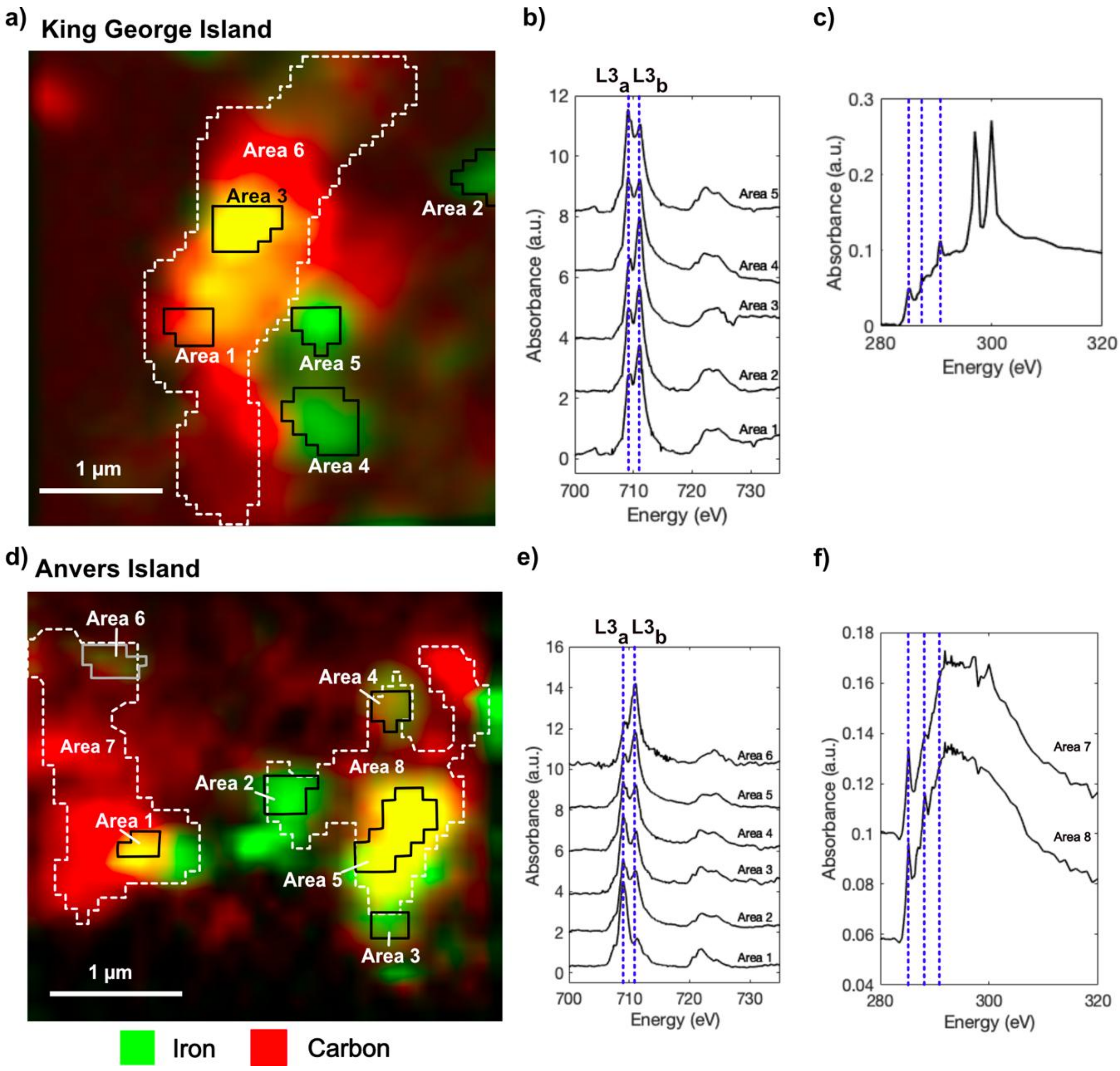

Supplementary Figure S2: Water column CTD sensor data collected inshore of the West Antarctic Peninsula. Temperature, salinity and potential density data from JR19002 (January 2020) plotted with a) depth, and b) optical transmission, Marian Cove, King George Island; c) depth, and d) optical transmission for Börgen Bay, Anvers Island; and e) depth, and f) optical transmission for Sheldon Cove, Adelaide Island. For the two marine-terminating systems, the Gade Line[1] is plotted on to indicate the temperature and salinity mixing line for subglacial meltwater.

51

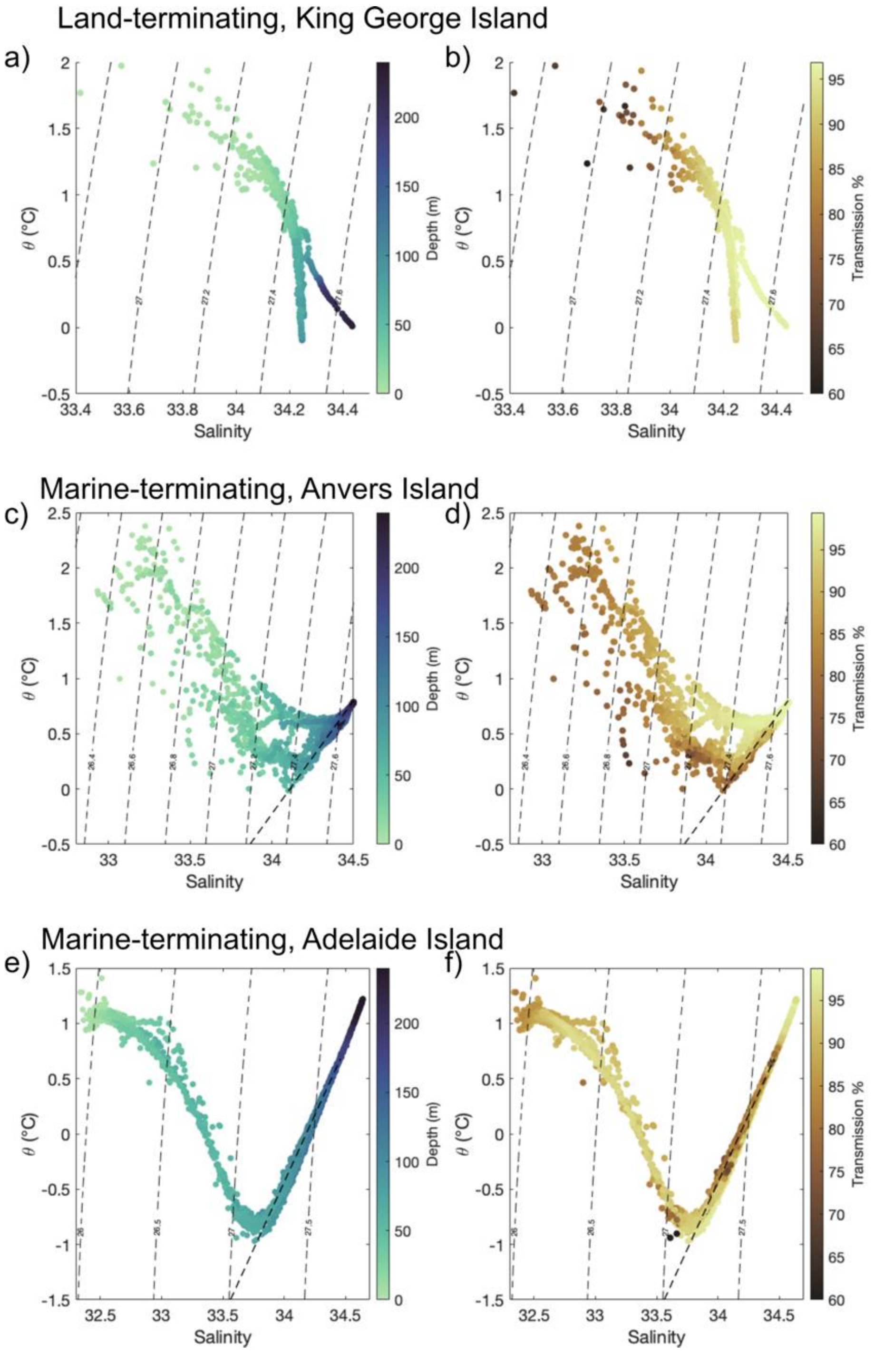

Land-terminating, King George Island
a)
b)
Marine-terminating, Anvers Island
c)
d)
Marine-terminating, Adelaide Island
e)
f)
θ (°C)
Salinity
Depth (m)
Transmission %

Supplementary Figure S3: Iron and Aluminium content of particles. Plotted Fe and Al wt %, measured using Scanning Electron Microscopy – Electron Dispersive Spectroscopy, from particles collected from the surface ocean of the three inshore WAP sites visited during January 2020. Error bars represent 1 s.d..

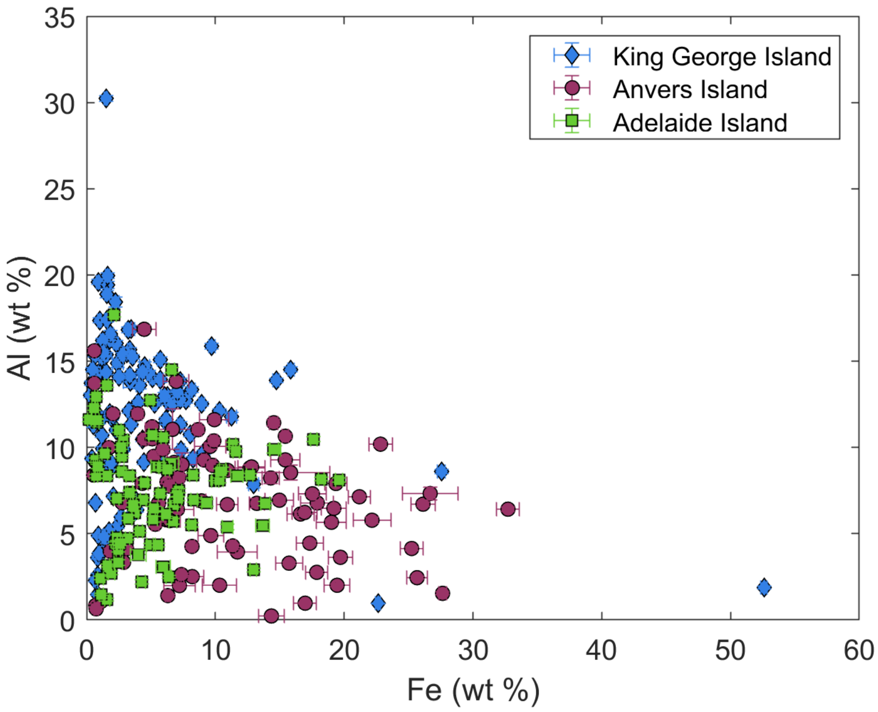

Supplementary Figure S4: Particulate manganese data across the West Antarctic Peninsula (WAP) shelf surface ocean. Particulate manganese (pMn) data collected during the Palmer Long Term Ecological Research (LTER) 2015 expedition, from the LTER grid across the WAP shelf, as shown in the main text Figure 4. Available surface ocean data for a) pMn with meteoric water contribution, modelled by the linear function pMn = $0.0724x - 0.13$ ($r^2 = 0.59$) and b) pMn with distance from the WAP coast (km) modelled by the linear function pMn = $-0.0010x + 0.22$ ($r^2 = 0.41$). Analytical precision on concentrations was better than $\pm$ 5%.

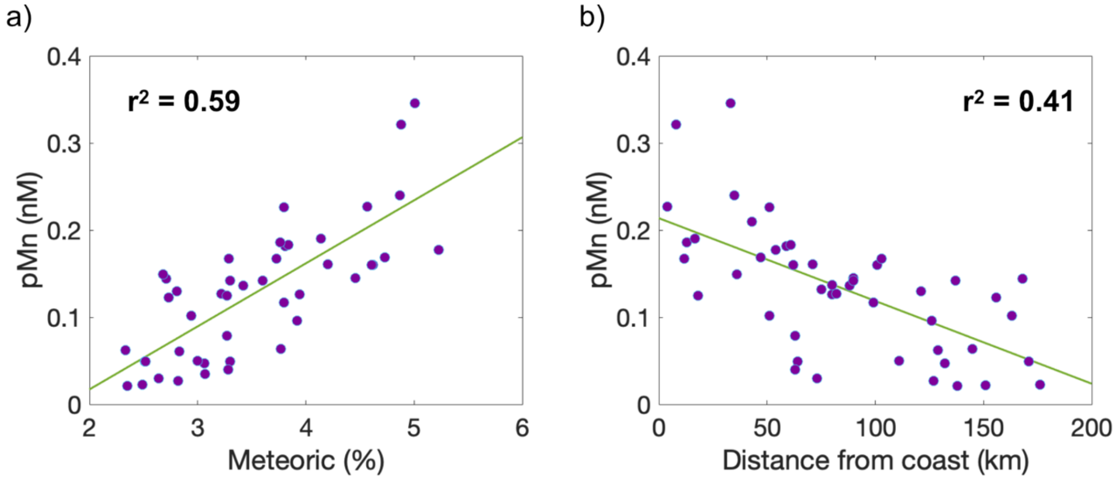

Supplementary Figure S5: **Analysis of Scanning X-ray Microscopy (SXM) beam damage of an iron-rich particle.** a) the particle, circled in white by the analysed pixel area, b) spectra of area circled in (a) over five dwell times, and c) the intensity ratio of peak $L_{3\text{-}a}$ and $L_{3\text{-}b}$, plotted with beam dwell time, indicating no discernible variability over time.

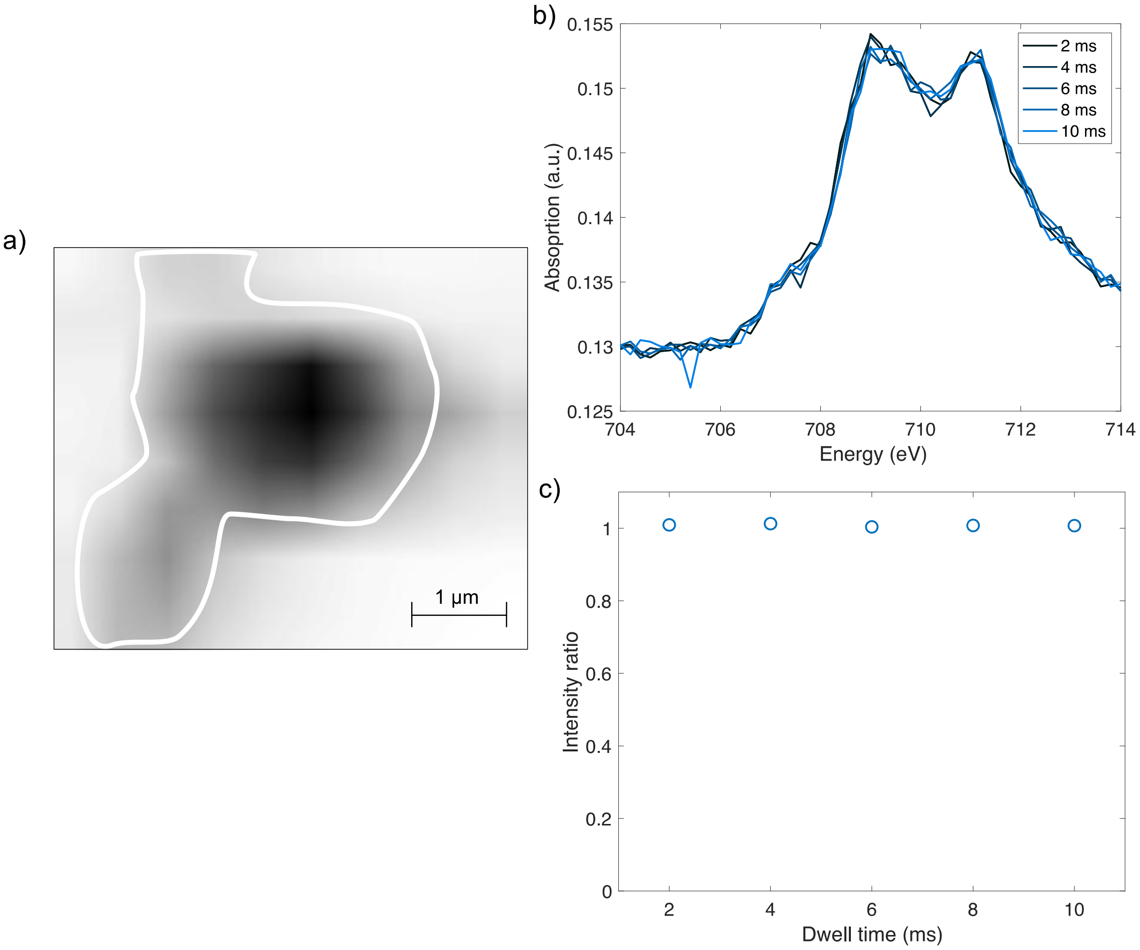

Supplementary Figure S6: Scanning Electron Microscopy – Electron Dispersive Spectroscopy (SEM-EDS) spectra and images showing elemental peaks from surface ocean particles. SEM-EDS analysis of expedition JR19002 surface ocean particles from a) – c) King George Island, d) – e) Anvers Island, and f) – i) Adelaide Island. Peaks in spectra represent presence of individual elements as labelled. For carbon (C) and gold (Au), abundance is removed from total composition due to the use of both elements in sample preparation. Spot size was approximately 1 µm, defined by the blue circles on each image. Units cps/eV refers to counts per second per electron volt, and unit keV refers to kilo-electron volts.

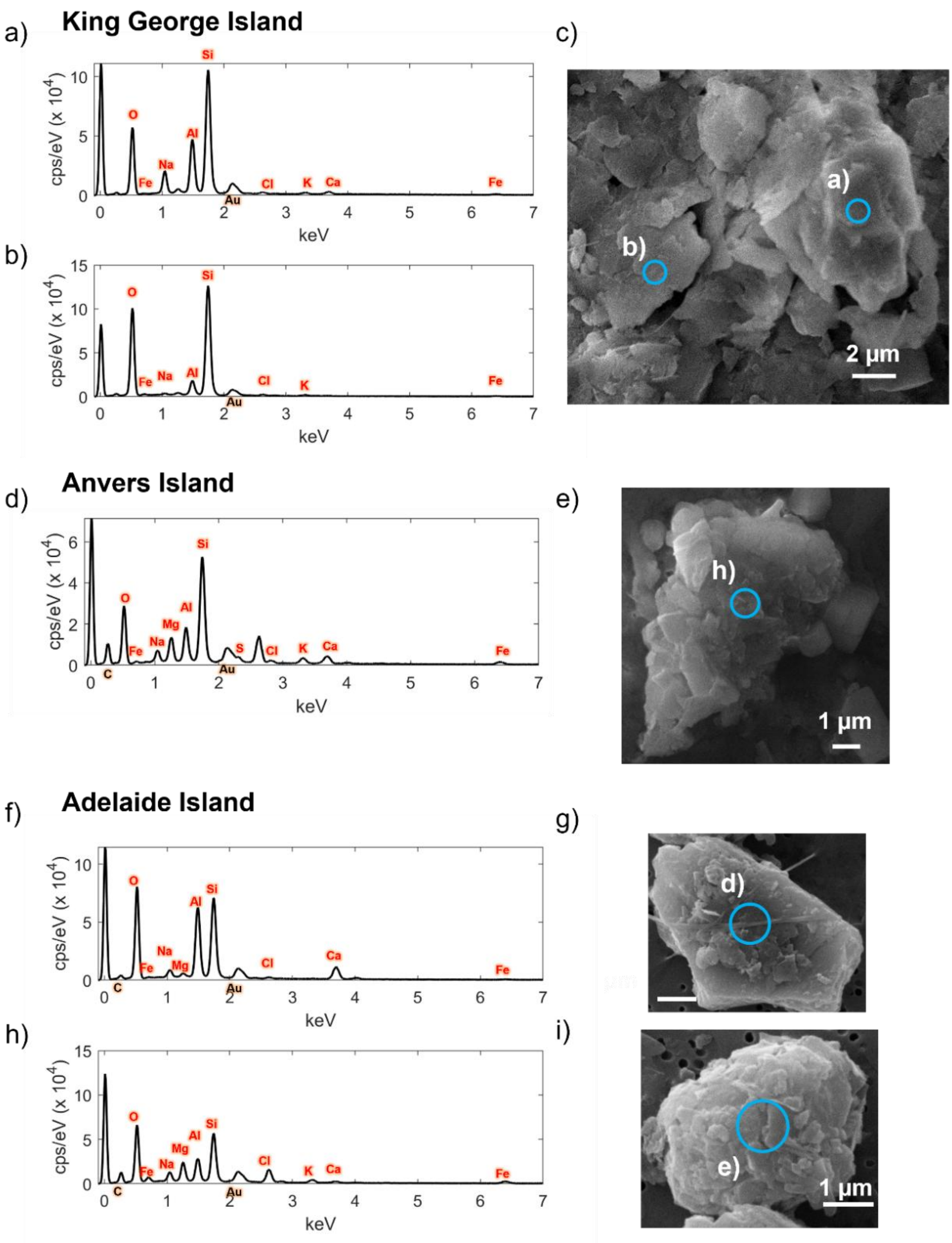
a)
King George Island
cps/eV (x 10^4)
keV
O
Na
Al
Si
Fe
Au
Cl
K
Ca
b)
c)
2 μm
d)
Anvers Island
C
Mg
S
e)
h)
1 μm
f)
Adelaide Island
g)
i)

Supplementary Figure S7: Modelled aggregate sinking depth as a function of total aggregate diameter and density. Sinking depth of a modelled aggregate/particle, as a function of total aggregate diameter and organic matter:mineral ratio, for an idealised spherical aggregate transported 160 km across the WAP shelf at an apparent transport rate of 0.01 ms^{-1}. The legend refers to the ratio of OM to mineral, where the density of OM is given as 1060 kg m^{-3} and the density of the mineral is given as 3960 kg m^{-3}. The grey line indicates the 40 m approximate mixed layer depth across the WAP shelf.

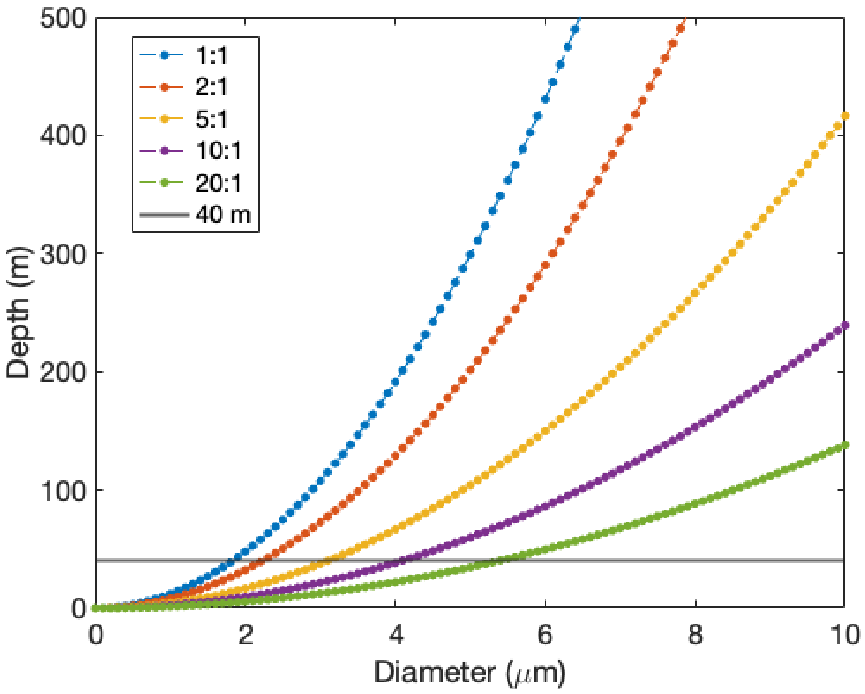

Supplementary Table S1: **Fe and C co-localisation analysis statistics for each analysed Scanning X-ray Microscopy image obtained from the three West Antarctic Peninsula sites, listed from north to south.**

Station	C file I.D.	Fe file I.D.	Pearsons R value
Marian Cove	75806	75981	0.37
Marian Cove	75806	75981	0.30
Marian Cove	75808	75983	n.c.*
Marian Cove	75810	75984	0.25
Marian Cove	75823	75965	0.52
Marian Cove	75825	75967	0.40
Marian Cove	75825	75967	n.c.
Marian Cove	75826	75968	0.44
Marian Cove	75827	75969	0.45
Marian Cove	75828	75970	0.39
Börgen Bay	75736	75938	n.c.
Börgen Bay	75739	75936	0.25
Börgen Bay	75740	75935	0.64
Börgen Bay	76008	76086	n.c.
Börgen Bay	76009	76087	0.02
Börgen Bay	76010	76088	n.c.
Börgen Bay	76011	76089	0.53
Börgen Bay	76012	76090	n.c.
Börgen Bay	76013	76091	0.42
Sheldon Cove	76035	76117	n.c.
Sheldon Cove	76036	76118	n.c.
Sheldon Cove	76037	76119	0.38
Sheldon Cove	76038	76120	n.c.
Sheldon Cove	76039	76121	n.c.
Sheldon Cove	75755	75750	0.53
Sheldon Cove	75754	75749	0.27
Sheldon Cove	75756	75951	0.68
Sheldon Cove	75757	75952	0.76
Sheldon Cove	75758	75953	0.44
Sheldon Cove	75759	75954	n.c.

*n.c. refers to not co-located, where Fe and C were not present in the same area.

Supplementary Table S2: List of sample and standard Fe X-ray Absorption Near Edge Structure spectra intensity ratio, ΔeV, and classification (Fe^{3+} /ΣFe) according to the estimation in Bourdelle et al., (2013)[2].

Sample	Proximity to glacier	L_{3-b} / L_{3-a}	ΔeV	Fe^{3+} /ΣFe	Reference
**Sheldon Cove**	Distal	0.7	2.1	25.19	**This study**
**Sheldon Cove**	Distal	1.3	1.8	57.15	**This study**
**Sheldon Cove**	Distal	1.2	1.5	49.37	**This study**
**Sheldon Cove**	Distal	1.8	1.6	83.39	**This study**
**Sheldon Cove**	Distal	1.4	1.6	58.54	**This study**
**Sheldon Cove**	Distal	1.0	2.3	41.46	**This study**
**Sheldon Cove**	Distal	1.3	2.0	55.13	**This study**
**Sheldon Cove**	Proximal	1.8	1.6	83.53	**This study**
**Sheldon Cove**	Proximal	1.9	1.2	86.46	**This study**
**Sheldon Cove**	Proximal	0.5	2.0	18.09	**This study**
**Sheldon Cove**	Proximal	2.2	1.6	101.99	**This study**
**Börgen Bay**	Distal	1.5	1.7	65.97	**This study**
**Börgen Bay**	Distal	1.9	1.5	87.88	**This study**
**Börgen Bay**	Distal	0.7	2.0	24.78	**This study**
**Börgen Bay**	Distal	1.5	2.1	63.82	**This study**
**Börgen Bay**	Distal	1.4	1.7	61.57	**This study**
**Börgen Bay**	Proximal	0.3	2.2	7.36	**This study**

**Börgen Bay**	Proximal	1.4	1.8	61.54	**This study**
**Börgen Bay**	Proximal	1.7	1.6	75.68	**This study**
**Börgen Bay**	Proximal	0.9	2.1	33.55	**This study**
**Börgen Bay**	Proximal	0.8	2.2	30.75	**This study**
**Börgen Bay**	Proximal	0.8	2.1	29.46	**This study**
**Börgen Bay**	Proximal	1.5	1.6	68.43	**This study**
**Börgen Bay**	Proximal	1.0	2.1	38.34	**This study**
**Börgen Bay**	Proximal	1.9	1.9	84.45	**This study**
**Börgen Bay**	Proximal	1.0	1.9	41.43	**This study**
**Börgen Bay**	Proximal	1.7	1.4	74.50	**This study**
**Börgen Bay**	Proximal	1.6	1.8	71.11	**This study**
**Börgen Bay**	Proximal	1.6	1.7	70.71	**This study**
**Börgen Bay**	Proximal	0.8	1.5	32.94	**This study**
**Börgen Bay**	Proximal	1.6	1.8	71.58	**This study**
**Börgen Bay**	Proximal	1.7	1.6	77.75	**This study**
**Börgen Bay**	Proximal	1.6	1.9	71.53	**This study**
**Marian Cove**	Distal	1.4	1.5	59.74	**This study**
**Marian Cove**	Distal	1.1	2.1	44.28	**This study**
**Marian Cove**	Distal	1.9	1.7	84.30	**This study**
**Marian Cove**	Distal	0.9	2.1	33.69	**This study**
**Marian Cove**	Distal	1.0	2.0	42.77	**This study**

**Marian Cove**	Distal	0.8	2.1	32.43	**This study**
**Marian Cove**	Distal	0.7	1.9	25.88	**This study**
**Marian Cove**	Distal	1.9	1.6	85.77	**This study**
**Marian Cove**	Distal	2.2	1.8	102.36	**This study**
**Marian Cove**	Distal	0.9	2.0	36.84	**This study**
**Marian Cove**	Distal	0.9	2.0	36.38	**This study**
**Marian Cove**	Distal	2.3	1.5	104.98	**This study**
**Marian Cove**	Proximal	1.0	1.7	38.66	**This study**
**Marian Cove**	Proximal	1.4	1.6	59.21	**This study**
**Marian Cove**	Proximal	1.3	1.6	54.61	**This study**
**Marian Cove**	Proximal	1.5	1.6	65.99	**This study**
**Marian Cove**	Proximal	1.0	2.0	39.60	**This study**
**Marian Cove**	Proximal	0.5	2.0	18.09	**This study**
**Marian Cove**	Proximal	0.5	2.0	13.81	**This study**
**Marian Cove**	Proximal	1.6	1.5	72.96	**This study**
**Marian Cove**	Proximal	1.5	1.5	67.55	**This study**
**Marian Cove**	Proximal	0.7	2.0	27.47	**This study**
**Marian Cove**	Proximal	0.7	2.0	27.19	**This study**
**Marian Cove**	Proximal	1.2	1.6	52.53	**This study**
**Marian Cove**	Proximal	1.8	1.6	79.53	**This study**
**Marian Cove**	Proximal	1.5	1.6	67.87	**This study**

**Marian Cove**	Proximal	2.2	1.7	99.73	**This study**
**Marian Cove**	Proximal	0.8	2.2	29.84	**This study**

Fe(III) standards

Amorphous Fe		0.505	1.496	90.08	[3]
Goethite		0.516	1.541	87.96	[3]
Hematite		0.663	1.525	66.38*	[3]
Lepidocrocite		0.477	1.653	95.92	[3]
Goethite		0.596	1.652	74.89*	[4]
Maghemite		0.554	1.427	81.28	[3]
Hematite		0.452	1.47	101.74	[5]
Akaganeite		0.472	1.54	97.03	[3]
Hematite		0.633	1.521	69.97*	[6]
Schwertmannite		0.689	1.413	63.52*	[7]
Ferrihydrite		0.698	1.409	62.58*	[8]
$FePO_4$		0.482	1.833	94.83	[9]
Ferrihydrite		0.485	1.502	94.18	[7]
Goethite		0.472	1.68	97.03	[10]
Ferrihydrite		0.486	1.48	93.97	[11]
Lepidocrocite		0.757	1.55	56.97*	[11]
$Fe_2(SO_4)_3$		0.424	1.67	109.08	[12]

Jarosite	0.388	1.85	120.07	[12]
$Fe_2(MO_4)_3$	0.376	1.551	124.20	[13]
**Fe(II)/(III) standards**				
Carbonate green rust	1.21	2.001	32.13	[10]
Green rust	1.284	1.276	29.74	[14]
Green rust intermediary	0.942	1.82	43.94	[10]
Catalyst 1	1.176	1.371	33.33	[6]
Catalyst 2	1.532	1.336	23.41	[6]
Catalyst 3	1.361	1.353	27.53	[6]
Magnetite	0.718	1.16	60.58	[3]
Magnetite	0.534	1.483	84.68	[15]
Magnetite	0.47	1.371	97.49	[16]
Magnetite	0.507	1.453	89.69	[17]
**Fe(II) Standards**				
Biotite	2.516	2.25	10.59	[11]
$Fe(II)Cl_2$	2.08	2.088	14.77	[15]
$Fe(II)PO_4$	1.976	2.139	16.04	[9]
$FeSiO_4$	4.876	2.74	0.92	[16]
Pyrite (partially oxidised)	1.037	1.167	39.06	[11]

Siderite	1.801	2.692	18.51	[11]
Pyrite (partially oxidised)	0.969	1.258	42.46	[8]
Pyrite	2.312	2	12.35	[12]
Fe(II)SO_4	3.058	2.39	7.05	[12]

Supplementary Table S3: Particulate and dissolved metal concentrations and meteoric water contribution for the West Antarctic Peninsula coastal sites (JR19002) presented in this study. KGI, AVI, and ADL refers to King George Island, Anvers Island, and Adelaide Island, respectively.

Station	Parameter[a]						
	pFe (nM)	pMn (nM)	pAl (μM)	pTi (nM)	dFe (nM)	dMn (nM)	Meteoric water (%)
**KGI-1***	370.1	13.4	2.2	15.9	3.5	33.3	4.2
**KGI-2***	272.9	9.2	1.4	12.7	4.2	33.7	5.7
**KGI-3**	575.4	20.7	3.1	25.7	3.2	35.1	7.9
**KGI-4**					6.4	54.0	5.41
**KGI-5**					3.2	34.9	3.9
**AVI-1***	78.0	2.5	0.23	5.2	7.9	5.4	6.6
**AVI-2**					5.5	5.3	5.8
**AVI-3***	142.9	4.3	0.44	9.8	7.2	6.5	5.6
**AVI-4**	27.8	0.8	0.07	2.0	4.4	3.2	7.4
**AVI-5**					5.9	6.4	3.1
**ADL-1***	29.1	1.0	0.11	2.1	3.3	3.3	6.1
**ADL-2***	38.9	1.3	0.15	3.0	3.2	3.1	6.2
**ADL-3**	51.8	1.9	1.9	3.9	3.4	3.1	4.7
**ADL-4**					4.4	3.7	6.7

[a] Analytical precision for particulate and dissolved metals was better than 5%. Uncertainties in final freshwater fractions are better than 1% for point values[18].

*At these stations, Fe and C speciation using XANES/NEXAFS is presented in this study.

Supplementary Table S4: Summary of elemental wt% in surface ocean particulates from the JR19002 expedition West Antarctic Peninsula sites. Determined using scanning electron microscopy – electron dispersive spectroscopy. Site samples are listed from north (King George Island) to central (Anvers Island) to south (Adelaide Island)

**Site**	**N**	**Si**		**Al**		**Mg**		**Na**		**Ca**		**Fe**	
		Wt %	*s.d.*	*Wt %*	*s.d.*	*Wt %*	*s.d.*	*Wt %*	*s.d.*	*Wt %*	*s.d.*	*Wt %*	*s.d.*
**KGI-1**	**33**	22.2	5.3	13.7	3.8	6.4	4.3	2.0	2.5	0.3	0.8	4.5	3.0
**KGI-2**	**34**	17.8	8.8	9.9	4.3	5.2	3.7	3.8	3.9	3.8	7.4	5.4	9.1
**KGI-3**	**37**	25.6	9.7	11.3	6.1	2.5	3.6	2.9	3.1	2.7	7.4	3.6	6.3
**AVI-1**	**34**	19.6	8.9	7.0	4.3	5.5	3.2	2.7	2.3	2.1	0.6	12.6	6.9
**AVI-2**	**31**	20.0	6.3	7.9	3.2	8.6	4.5	5.1	4.7	1.5	0.3	5.8	3.0
**AVI-3**	**32**	14.5	8.1	5.9	3.3	7.2	7.8	7.2	7.8	2.1	0.6	10.2	8.0
**ADL-1**	**29**	27.2	8.1	7.8	3.4	2.9	2.8	3.7	3.3	2.6	3.5	4.4	4.9
**ADL-2**	**30**	17.2	6.2	7.4	2.2	5.3	3.1	7.7	3.9	1.9	1.9	4.8	4.9
**ADL-3**	**29**	19.3	6.7	7.9	3.9	4.7	3.9	7.0	4.0	3.4	6.0	4.7	4.6
**KGI AVG**	**104**	22.0	8.8	11.6	5.0	4.6	4.1	2.9	3.3	2.3	7.0	4.5	6.6
**AVI AVG**	**97**	17.7	8.4	6.6	3.9	7.2	4.1	3.1	6.9	1.9	6.4	8.1	7.6
**ADL AVG**	**88**	18.5	8.6	7.8	3.3	4.5	3.4	6.4	5.8	1.7	4.0	4.0	4.5

*Wt % refers to the mean wt %, normalised to the total number of particles. S.d. refers to 1 standard deviation of the total sample set.

KGI AVG, AVI AVG and ADL AVG refers to the mean wt % of the three sites, with s.d. referring to the standard deviation between site values.

Supplementary Table S5: Station names and coordinates for West Antarctic Peninsula JR19002 sampling sites, with the corresponding parameters presented in this study listed.

Station	Expedition ID*	Latitude	Longitude	Parameter
KGI-1	MC1	-62.2100	-58.7592	XANES/NEXAFS, pTM, dFe, dMn, salinity, $\delta^{18}O$, SEM-EDS, DOC
KGI-2	MCC	-62.2031	-58.7396	XANES/NEXAFS, pTM, dFe, dMn, salinity, $\delta^{18}O$, SEM-EDS, DOC
KGI-3	MCE	-62.2036	-58.7423	pTM, dFe, dMn, salinity, $\delta^{18}O$, SEM-EDS, DOC
KGI-4	MCA	-62.2111	-58.7542	dFe, dMn, salinity, $\delta^{18}O$, DOC
KGI-5	MCD	-62.2036	-58.7349	dFe, dMn, salinity, $\delta^{18}O$, DOC
AVI-1	BB0	-64.7651	-63.4653	XANES/NEXAFS, dFe, dMn, salinity, $\delta^{18}O$, SEM-EDS, DOC
AVI-2	BBC	-64.7053	-63.4654	pTM, dFe, dMn, salinity, $\delta^{18}O$, SEM-EDS, DOC
AVI-3	BBE	-64.7034	-63.4552	XANES/NEXAFS, pTM, dFe, dMn, salinity, $\delta^{18}O$, SEM-EDS, DOC
AVI-4	BBA	-64.7313	-63.4670	dFe, dMn, salinity, $\delta^{18}O$, DOC
AVI-5	BBD	-64.7072	-63.4654	dFe, dMn, salinity, $\delta^{18}O$, DOC
ADL-1	SCA	-67.5417	-68.2768	XANES/NEXAFS, pTM, dFe, dMn, salinity, $\delta^{18}O$, SEM-EDS, DOC
ADL-2	SCE	-67.5139	-68.2434	XANES/NEXAFS, pTM, dFe, dMn, salinity, $\delta^{18}O$, SEM-EDS, DOC
ADL-3	SC6	-67.5151	-68.2460	pTM, dFe, dMn, salinity, $\delta^{18}O$, SEM-EDS, DOC
ADL-4	SC2	-67.5493	-68.2699	dFe, dMn, salinity, $\delta^{18}O$

*Station names provided in this study are adjusted to assist with clarity from those given in the JR19002 cruise report.

Supplementary Table S6: Endmember values for salinity and stable oxygen isotopes ($\delta^{18}O$) used to calculate meteoric water contribution. For the WAP inshore bays, deconvolution of sea ice melt, meteoric water, and modified Circumpolar Deep Water uses the following endmember salinity and $\delta^{18}O$ values, as previously published in [18]. The abbreviation mCDW refers to modified Circumpolar Deep Water.

	Adelaide Island	Anvers Island	King George Island	Antarctic Peninsula shelf
Salinity				
Sea ice melt	7.0	7.0	5.0	7.0
Meteoric water	0.0	0.0	0.0	0.0
mCDW	34.62	34.65	34.40	34.73
***$\delta^{18}O$* (‰)**				
Sea ice melt	2.1	1.1	1.6	2.1
Meteoric water	-16	-12	-11	-16
mCDW	0.04	0.0	-0.2	0.1

Supplementary Note 1:

Calculation of particle settling rate using Stokes' Settling Law

In this study, we find that iron (Fe) is present as both isolated particles and aggregated with organic carbon-rich material. The simultaneous export away from the coast, and potential retention of these particles and aggregates within the mixed layer, as discussed in this study, will be partly a function of the material settling velocity. In Figure 3, it is demonstrated that carbon (C) and Fe-rich aggregated material could range from around 1 – 10 µm, yet isolated Fe-rich particle diameters are estimated as 0.2 – 1 µm. Fe-rich aluminosilicate minerals, pyrite, or iron (oxyhydr)oxides [19], have a different density (3090 – 5000 kg m^{-3}) to organic matter (OM) (~1060 kg m^{-3}) [20]. Assuming the particle or aggregate remains intact, the settling velocity is predominantly a function of diameter and average material density, and therefore the material observed in this study will have a range of settling velocities.

To calculate the settling velocity for a spherical particle under laminar flow using Stokes' Law, we adopted the following equation[21]:

$$V = g\ D^2 * \frac{(d_p - d_m)}{18v} \quad (1)$$

V: settling velocity (m s^{-1})

G: gravitational acceleration, 9.8 m s^{-2}

D: particle diameter (m)

d_p: density of particle

d_m: density of medium, 1029 kg m^{-3}

v: viscosity of seawater, 1.88×10^{-3} kg m^{-1} s^{-1}

For particle density, we use a range of values based on the reported density of the relevant minerals. For a spherical particle of pure biotite, of d_p = 3090 km m^{-3} and 0.2 µm diameter, the calculated settling velocity is 2.39 × 10^{-8} m s^{-1} or 0.75 m yr^{-1}. For a pyrite particle of d_p = 5000 kg m^{-3} and 1 µm diameter, the calculated settling velocity is 1.15 × 10^{-6} m s^{-1} or 36 m yr^{-1}. The density of the common Fe (oxyhydr)oxides ferrihydrite and goethite are estimated as 3960 and 4280 kg m^{-3}, respectively [19] . The range for Fe-rich particle types of 0.2 – 1 µm with a d_p range of 3090 – 5000 kg m^{-3} is therefore 0.75 – 36 m yr^{-1} or 0.38 – 18 m in 6 months. Isolated particles of <1 µm are therefore retained in the top 40 m over a 6-month period.

All particle density values are taken from mindat.org unless otherwise referenced.

Calculating the aggregate settling velocity of an Fe-C-rich aggregate using Stokes' Settling Law

To calculate the settling velocity of an Fe- and C-rich aggregate such as those observed in this study (Figure 3), requires estimating the average aggregate density and diameter. Average aggregate density is calculated using a range of OM:mineral volume ratios of 1, 2, 5, 10 and 20. Aggregate diameter of 1, 2, 5 and 10 µm is estimated, using the examples in Figure 3. Taking the average density and diameter, we again use Stokes' Settling Law for a spherical aggregate. For an aggregate of OM:ferrihydrite of 1:1, and a diameter of 10 µm, an aggregate would have a settling velocity of 7.45 × 10^{-5} m s^{-1}, or 2350 m yr^{-1}. For an aggregate of OM:ferrihydrite of 20:1 and a diameter of 1 µm, the settling velocity is 8.15 × 10^{-8} m s^{-1} or 2.6 m yr^{-1}.

Application to the WAP shelf

The summer mixed layer depth towards the shelf break at the WAP generally ranges between 30 and 40 m [22,23]. Applying the Stokes settling calculations, combined with a conservative across-shelf effective transport rate of 0.01 ms^{-1}, the depth to which an isolated particle of 0.2 – 1 µm and a density of 3090 – 5000 kg m^{-3} would be 0.38

– 18 m in a 6-month period, or the time taken to travel 160 km. For aggregates of Fe-rich particles and C-rich organic matter ranging from 1 – 10 µm, the sinking rate is more variable, due to the influence of both diameter and average density upon the total aggregate. Imposing the same across-shelf effective transport rate of 0.01 m s^{-1}, aggregates of < 2 µm would mostly be retained in the top 40 m over a 6-month period. Aggregates 2 – 5 µm may be retained in the top 40 m, and most aggregates >5 µm would sink below 40 m. Assuming the mineral density is 3960 kg m^{-3}, and the OM:mineral ratio varies from 1 – 20, the relationship for aggregate diameter with sinking depth is demonstrated in **Supplementary Figure S7** for a 6-month period (160 km lateral transport). Therefore, we hypothesise that many aggregates up to 5 µm diameter would be retained in the top 40 m, and an increased organic matter proportion relative to mineral would increase this retention potential.

Supplementary References

1. Gade, H. G. Melting of ice in sea water: A primitive model with application to the Antarctic ice shelf and icebergs. *Journal of Physical Oceanography* **9**, 189-198 (1979).
2. Bourdelle, F. *et al.* Quantification of the ferric/ferrous iron ratio in silicates by scanning transmission X-ray microscopy at the Fe L 2, 3 edges. *Contributions to Mineralogy and Petrology* **166**, 423-434 (2013).
3. Von der Heyden, B., Roychoudhury, A., Mtshali, T., Tyliszczak, T. & Myneni, S. C. B. Chemically and geographically distinct solid-phase iron pools in the Southern Ocean. *Science* **338**, 1199-1201 (2012).
4. Kneedler, E., Rothe, J., Weissmahr, K., Pecher, K. & Tonner, B. Identification of green rust in environmental compounds using XANES of Fe-LII, III edges. *Advanced Light Source Annual Compendium of Abstracts* (1997).
5. Bluhm, H. *et al.* Soft X-ray microscopy and spectroscopy at the molecular environmental science beamline at the Advanced Light Source. *Journal of Electron Spectroscopy and Related Phenomena* **150**, 86-104 (2006).
6. de Smit, E. *et al.* Nanoscale chemical imaging of a working catalyst by scanning transmission X-ray microscopy. *Nature* **456**, 222-225 (2008).
7. Chan, C. S., Fakra, S. C., Edwards, D. C., Emerson, D. & Banfield, J. F. Iron oxyhydroxide mineralization on microbial extracellular polysaccharides. *Geochimica et Cosmochimica Acta* **73**, 3807-3818 (2009).
8. Hawkings, J. R. *et al.* Biolabile ferrous iron bearing nanoparticles in glacial sediments. *Earth and Planetary Science Letters* **493**, 92-101 (2018).
9. Miot, J. *et al.* Extracellular iron biomineralization by photoautotrophic iron-oxidizing bacteria. *Applied and environmental microbiology* **75**, 5586-5591 (2009).
10. Pantke, C. *et al.* Green rust formation during Fe (II) oxidation by the nitrate-reducing Acidovorax sp. strain BoFeN1. *Environmental science & technology* **46**, 1439-1446 (2012).
11. Toner, B. M. *et al.* Preservation of iron (II) by carbon-rich matrices in a hydrothermal plume. *Nature Geoscience* **2**, 197-201 (2009).
12. Liu, H.-c. *et al.* Iron L-edge and sulfur K-edge XANES spectroscopy analysis of pyrite leached by Acidianus manzaensis. *Transactions of Nonferrous Metals Society of China* **25**, 2407-2414 (2015).
13. Shirakawa, J., Nakayama, M., Wakihara, M. & Uchimoto, Y. Changes in electronic structure upon lithium insertion into Fe2 (SO4) 3 and Fe2 (MoO4) 3 investigated by X-ray absorption spectroscopy. *The Journal of Physical Chemistry B* **111**, 1424-1430 (2007).
14. Christiansen, B. C., Balic-Zunic, T., Dideriksen, K. & Stipp, S. L. S. Identification of Green Rust in Groundwater. *Environmental Science & Technology* **43**, 3436-3441 (2009). https://doi.org:10.1021/es8011047

15 Dynes, J. J. *et al.* Speciation and quantitative mapping of metal species in microbial biofilms using scanning transmission X-ray microscopy. *Environmental science & technology* **40**, 1556-1565 (2006).

16 De Groot, F. M. in *Journal of Physics: Conference Series.* 012004 (IOP Publishing).

17 De Castro, A. *et al.* L-edge inner shell spectroscopy of nanostructured Fe3O4. *Journal of magnetism and magnetic materials* **233**, 69-73 (2001).

18 Jones, R. L. *et al.* Continued glacial retreat linked to changing macronutrient supply along the West Antarctic Peninsula. *Marine Chemistry* **251**, 104230 (2023).

19 Jansen, E., Kyek, A., Schäfer, W. & Schwertmann, U. The structure of six-line ferrihydrite. *Applied Physics A* **74**, s1004-s1006 (2002).

20 Laurenceau-Cornec, E. C. *et al.* New guidelines for the application of Stokes' models to the sinking velocity of marine aggregates. *Limnology and Oceanography* **65**, 1264-1285 (2020).

21 Schwarzenbach, R. P., Gschwend, P. M. & Imboden, D. M. *Environmental organic chemistry*. (John Wiley & Sons, 2016).

22 Carvalho, F., Kohut, J., Oliver, M. J. & Schofield, O. Defining the ecologically relevant mixed-layer depth for Antarctica's coastal seas. *Geophysical Research Letters* **44**, 338-345 (2017).

23 Vernet, M. *et al.* Primary production within the sea-ice zone west of the Antarctic Peninsula: I—Sea ice, summer mixed layer, and irradiance. *Deep Sea Research Part II: Topical Studies in Oceanography* **55**, 2068-2085 (2008).
